# Stroke and Fabry Disease: A Review of Literature

**DOI:** 10.7759/cureus.12083

**Published:** 2020-12-14

**Authors:** Vinayak Mishra, Amit Banerjee, Arohi B Gandhi, Ifrah Kaleem, Josh Alexander, Mohamed Hisbulla, Vishmita Kannichamy, Sharathshiva Valaiyaduppu Subas, Pousette Hamid

**Affiliations:** 1 Internal Medicine, California Institute of Behavioral Neurosciences and Psychology, Fairfield, USA; 2 General Medicine, California Institute of Behavioral Neurosciences and Psychology, Fairfield, USA; 3 Neurology, California Institute of Behavioral Neurosciences and Psychology, Fairfield, USA

**Keywords:** stroke, fabry disease, cerebrovascular disease, pediatric stroke

## Abstract

Fabry disease is an X-linked lysosomal storage disorder caused by a mutation in the alpha-galactosidase A (GLA) gene, leading to the deficiency of alpha-galactosidase A enzyme. The natural history of the affected patients (both males and females) includes neurovascular complications, such as cerebrovascular disease at a relatively young age. The pathophysiology behind the vascular involvement is primarily attributed to the accumulation of globotriaosylceramide and its derivatives in the vascular endothelium and vascular smooth muscle cells. MRI is the gold standard radiological investigation to detect the white matter lesions characteristic of Fabry disease's neurological involvement. More studies should focus on the utility of universally screening patients with young stroke for Fabry disease and the effectiveness of enzyme replacement therapy to prevent stroke. This review offers a synopsis of the current knowledge of the pathophysiology, neuroradiology, treatment, and prognosis of cerebrovascular disease in Fabry patients.

## Introduction and background

In 1898, Anderson and Fabry independently described patients with skin manifestations of red and purple maculopapular lesions [1-4]. Fabry disease was initially described as a dermatological disorder, called "Angiokeratoma Corporis Diffusum," in various case reports [5,6]. Later, Sweeley and Klionsky recognized this condition as an X-linked multisystemic lipid storage disorder [7]. They discovered two types of abnormal glycolipids on microscopic and biochemical analysis of kidney samples from a patient who suffered from Fabry disease's characteristic features and died of renal failure [7]. The mutation of the alpha-galactosidase A (GLA) gene, which is present on the X chromosome's long arm and encodes the alpha-galactosidase A enzyme, causes the disease [8]. The alpha-galactosidase A enzyme's decreased activity causes pathological accumulation of globotriaosylceramide (GL-3) in lysosomes of various cells throughout the body [9]. This deposition begins in the fetal stage, as demonstrated by examining the placenta [10,11].

Fabry disease affects people throughout the world, irrespective of ethnicity and nationality. Given the absence of a comprehensive international database for this rare disease, it is challenging to estimate the true prevalence of this condition. Several studies have reported the prevalence of this disease, ranging from 1 in 476,000 to 1 in 117,000 by estimating the birth prevalence and neonatal screening for the specific mutations [12-15].

Cerebrovascular disease is a frequent manifestation, with the frequency of stroke in young men (aged 25-44 years) almost 12 times higher than the general population and ten times higher prevalence in young women [16]. Sims et al. collected data from the Fabry Registry and found that 6.9% of males and 4.3% of females developed stroke [17]. The mechanism and pathophysiology of stroke, in this case, is yet to be elucidated. Besides, the role of enzyme replacement therapy in preventing cerebrovascular disease is undetermined; given the lack of strong evidence.

This article reviews the current literature on the mechanism, neuroimaging, management, and prognosis of cerebrovascular disease in the case of Fabry disease. We aim to highlight the importance of suspecting this disease in cases of "young stroke," explain the role of neuroimaging in early detection of vascular lesions, and describe ways to prevent neurovascular complications.

## Review

Pathophysiology of cerebrovascular disease in Fabry disease

The mechanism of stroke in Fabry disease is still uncertain. Enhanced knowledge about the mechanism of this disease can help us develop effective therapy; and elucidate the role of additional treatment modalities - antiplatelets, lipid-lowering agents, and inhibitors of the renin-angiotensin-aldosterone system in the mitigation of vascular dysfunction [18].

**Figure 1:**
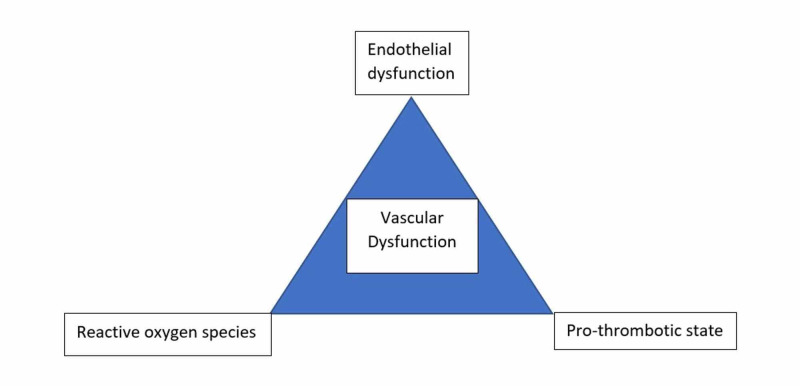
Triad of vascular dysfunction in Fabry disease

Cerebral vasculopathy in Fabry disease involves both large vessels and small vessels [19]. Researchers have hypothesized that endothelial dysfunction, cerebral hyper-perfusion, pro-thrombotic state, and higher synthesis of reactive oxygen species contribute to the vascular dysfunction in Fabry disease [19]. The evolving application of enzyme replacement therapy (ERT) has highlighted that eliminating accumulated glycosphingolipids from the endothelial cells does not prevent the progression of vascular damage (Figure 1) [19].

The abnormal accumulation of glycosphingolipids within endothelial cells might play a role in the smooth muscle proliferation in the arterial media layer that culminates in hydrodynamic damage to the arterial wall [18,20]. Barbey et al. demonstrated left ventricular hypertrophy and increased intima-media thickness of the common carotid artery in patients with Fabry disease, which was proved to be due to a circulating growth factor in an in-vitro study [21]. This increased arterial thickness was observed equally in both male and female patients despite different plasma alpha-galactosidase enzyme activity [21]. Thereby, we can conclude that the arterial remodeling in Fabry disease occurs irrespective of the residual enzyme activity level. The quantity of glycosphingolipid deposition does not explain vascular dysfunction.

Atherosclerosis can worsen the vasculopathy of Fabry disease. It disturbs the hemodynamic balance by further altering the intima-media thickness of arterial walls. Irrespective of its etiology, chronic kidney disease worsens, and accelerates atherosclerosis in the circulatory system [22]. In Fabry disease, advanced renal involvement heralds a worse prognosis and lower response to ERT; this could be due to the accelerated atherosclerosis in this case [23-25]. Besides, researchers have shown that the clearance of accumulated glycosphingolipids (as evidenced by histopathological examination) by ERT in Fabry patients with advanced renal disease did not hinder the progression of the disease [23-25].

Barbey et al. highlighted the role of a growth-promoting factor in smooth muscle proliferation in Fabry arteries in an in-vitro study [21]. This smooth muscle hypertrophy plays the most crucial role in increasing the arterial intima-media thickness. The discovery of this growth-promoting factor will have significant implications for the treatment of vasculopathy. Brakch et al. first used a biochemical method to identify sphingosine-1-phosphate as the growth-promoting factor present in Fabry blood plasma and then used an experimental mice model to confirm this [26]. Similarly, Aerts et al. described higher concentrations of deacetylated globotriaosylceramide, called globotriaosylsphingosine or lyso-Gb3, in the plasma of Fabry disease patients [27]. In an in-vitro study-lyso-Gb3 (at such high concentrations) increased the pathological accumulation of globotriaosylceramide and triggered the proliferation of vascular smooth muscle cells [27].

**Figure 2:**
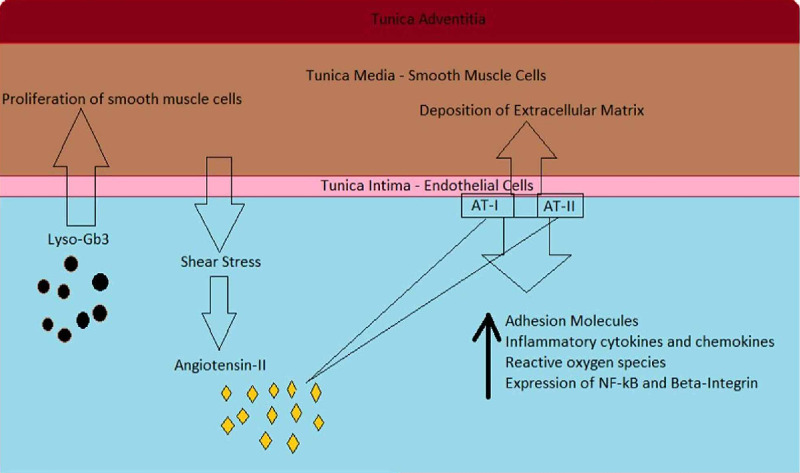
Role of lyso-Gb3 and angiotensin-II in arterial remodeling AT-I: angiotensin-I receptor; AT-II: angiotensin-II receptor

Smooth muscle cells in this condition turn out to be guilty by association. The abnormally proliferating vascular smooth muscle cells express adhesion molecules and produce cytokines that lead to the influx of inflammatory cells such as monocytes and lymphocytes and initiates the cascade of inflammation in the vessel wall [28]. Arterial remodeling results in thickened, poorly compliant vascular walls, which in association with hyperdynamic circulation, can increase angiotensin-II levels by triggering the local renin-angiotensin system. Higher levels of angiotensin-II further heighten adhesion molecules' expression and the production of inflammatory cytokines and chemokines [29,30]. This has a pro-inflammatory effect on leukocytes, vascular smooth muscles, and endothelial cells in the milieu [18,29,30]. Angiotensin-II's action on the AT1 receptor leads to increased production of reactive oxygen species, expression of NF-kB, and increased expression of β-integrin [29,30]. The raised expression of β-integrin abnormally elevates the extracellular matrix's deposition in the vessel wall, further decreasing its compliance. This inflammatory process, along with oxidative stress, weakens the vessel wall as it activates protease-mediated extracellular matrix degradation and apoptosis of smooth muscle cells [31]. The progressive weakening of the vessel wall culminates in dilatation and aneurysm formation (Figure 2) [31]. 

Neuroimaging in Fabry disease

MRI is considered the gold standard imaging modality to detect cerebrovascular involvement in Fabry disease. It facilitates precise evaluation of the pattern and severity of brain damage in Fabry patients with focal neurological deficits [32]. Several advanced imaging methods (diffusion tensor imaging, functional MRI, MRI spectroscopy, and Positron emission tomography) have been utilized to improve our understanding of the mechanisms involved in the pathogenesis of cerebrovascular disease in Fabry disease [32]. Such advanced neuroimaging techniques have helped broaden our perspective beyond the major cerebrovascular events in this complex condition. 

Fabry disease has non-specific neuroimaging findings, which mainly include white matter hyperintensities in the periventricular and subcortical areas and posterior circulation vascular anomalies. Since a significant number of people with this condition experience their first cerebrovascular event before the diagnosis [33], stroke physicians must be aware of the imaging features of this rare lysosomal storage disease.

Ectasia and elongation of the basilar artery are a frequent, albeit inconsistent neuro-radiological feature of Fabry disease [32]. The assessment of basilar artery diameter in young stroke patients could be used to detect Fabry disease as patients with this disease have higher basilar artery diameters than those not with this disease [34]. In a comparative study performed by Fellgiebel et al., the estimation of basilar artery diameter was more accurate than other imaging parameters to detect Fabry disease, especially in cryptogenic stroke cases [35]. Time-of-flight magnetic resonance angiography (MRA) can be used to evaluate the elongation, tortuosity, and diffuse (or focal) aneurysmal dilatations of the basilar artery [36]. This increase in basilar artery diameter is higher in males than females: most probably due to the X-linked inheritance of this lysosomal storage disorder [36].

White matter lesions in Fabry disease are most likely due to cerebral microangiopathy; most patients exhibit these without any clinical neurological deficit [37]. Quantification of white matter hyperintensity with volumetric MRI techniques can help evaluate disease burden, progression, and even improvement after initiation of enzyme replacement therapy [38,39]. In the future, this may serve as a tool to judge the effectiveness of novel treatments. White matter hyperintensity on MRI may be due to small vessel microangiopathy as its presence correlates with the risk of stroke, poor post-stroke outcome, and cognitive disability in ageing adults [39]. In this case, also, the white matter hyperintensity volume is higher in males than females [39]. Determinants of white matter hyperintensity quantity are the age of the patient, prior history of cerebrovascular disease, and presence of Fabry disease-related heart (cardiomyopathy, cardiac arrhythmia), kidney (proteinuria), and gastrointestinal problems [39]. 

 Diffusion tensor imaging is a new MRI technique that measures water diffusion to evaluate the brain's white matter organization. It is a useful method to measure the microstructural white matter changes, especially in the periventricular region, in patients with Fabry disease [40]. Besides, it can also be used to follow-up the cerebral effects in patients who develop complications and those who are started on enzyme replacement therapy [41]. Interestingly, the diffusion tensor imaging findings did not differ between male and female patients [41]. Region of interest (ROI) based analysis of the findings from diffusion tensor imaging demonstrates that the diffusivity changes in some areas of the brain can occur independently of the presence of white matter hyperintensity; besides, these changes can be interpreted as signs of early cerebral vasculopathy [41].

Pulvinar sign is a "highly specific" sign of Fabry disease; it is almost exclusively found in male patients with coexisting severe cardiac and renal involvement [42]. It is described as - "bilaterally increased signal intensity of the pulvinar region (posterior thalamus) on T1-weighted images" [42]. This is hypothesized to be due to regional hyper-perfusion that leads to microvascular calcifications [43]. This is probably an irreversible finding since it did not recede on starting enzyme replacement therapy when followed up for an average of three years [42].

Treatment and prognosis of cerebrovascular disease in Fabry disease

In 2011, the American Heart Association/American Stroke Association recommended alpha-galactosidase enzyme replacement therapy for prevention of stroke in patients with ischaemic stroke or transient ischaemic attack with Fabry disease (class I; level B evidence); however, this recommendation was removed in the subsequent 2014 edition of the guidelines given "the rarity and specialized nature of this condition" [44]. Although enzyme replacement therapy (alpha-galactosidase A) has been shown to improve cerebral vasculopathy, it has not decreased stroke incidence in patients with Fabry disease [45].

In a case report by Yamadera et al. - in a 27-year-old male patient with Fabry disease who had widespread white matter lesions, enzyme replacement therapy, comprising of biweekly infusions of alpha-galactosidase beta (1 mg/kg), for 12 months led to the disappearance of a majority of the white matter lesions; along with improvement in the cell counts and protein levels in the cerebrospinal fluid [46]. This suggests that enzyme replacement therapy might have a role in ameliorating cerebral vasculopathy in Fabry disease.

In a clinical study on eight patients with Fabry disease who took enzyme replacement therapy (agalsidase-alfa, 0.2 mg/kg every week) for two years and were serially followed during this period: there were mixed results; white matter lesions stayed stable in three patients, worsened in one patient, and diminished only in one patient [47]. It was uncertain whether these findings were a part of the natural history of Fabry disease or were influenced by enzyme replacement therapy.

Antiplatelet agents such as aspirin, clopidogrel, or ticlopidine are also recommended to prevent stroke and transient ischaemic attack in Fabry disease [48]. Proper control of blood pressure and adequate hydration are also advised to maintain optimum cerebral perfusion [48].

Alpha-galactosidase, A enzyme replacement therapy, has been shown to decrease the severity of neuropathic pain, improve glomerular filtration rate, reverse the pathology in glomerular architecture, and reduce the QRS-complex duration when administered intravenously at a dose of 0.2 mg/kg every 15 days for six months in patients with Fabry disease [49]. 

Migalastat, an oral pharmacologic chaperone, stabilizes specific mutated forms of the alpha-galactosidase enzyme and increases enzyme activity [50]. In a randomized, placebo-controlled, double-blinded trial, patients treated with migalastat had lower levels of globotriaosylceramide in the plasma and a reduced number of globotriaosylceramide inclusions in the renal parenchyma compared with the placebo group after six months [50]. However, the aforementioned results are derived from secondary analyses of only those patients with migalastat-amenable alpha-galactosidase enzyme mutations. 

Limitations

The treatment and prognosis section of this review article lacks randomized controlled trials and systematic reviews with meta-analyses. Besides, it also lacks critical data appraisal and appropriate data synthesis. Therefore, this review does not expand the body of knowledge concerned with the management of cerebrovascular disease in Fabry disease.

## Conclusions

Fabry disease is an important cause of young stroke and cryptogenic stroke, despite its rarity. Therefore, physicians, internists, neurologists, and pediatricians should be aware of this condition and its multisystemic manifestations. This will facilitate earlier diagnosis and initiation of the necessary treatment, including enzyme replacement therapy, given that it is more effective if started at an earlier age. Focused screening for Fabry disease in all the young stroke cases might be routinely indicated in the future. The unreliability of enzyme replacement therapy in reducing stroke incidence emphasizes the need for pertinent long-term observational studies and well-designed randomized clinical trials.

Further research, primarily randomized controlled trials, is warranted in the Fabry cerebral vasculopathy domain to design better treatment protocols with improved neurological outcomes. Cerebrovascular attacks can occur in Fabry disease patients "silently" without any impending warning signs, and even before the diagnosis of the condition. Therefore, additional studies must be dedicated to finding biomarkers or neuroimaging features that can detect and diagnose neurovascular pathology and monitor its progression; to enable us to estimate the risk of stroke in specific cases.
